# Pediatric and adult primary human airway epithelia exhibit similar rhinovirus C replication, innate responses, and expression of protein receptor

**DOI:** 10.1128/spectrum.01611-26

**Published:** 2026-08-31

**Authors:** Lorellin A. Durnell-Bettis, Ian R. Taylor, You Li, Scott H. Randell, Yury A. Bochkov, James E. Gern, Matthew R. Vogt

**Affiliations:** 1 Department of Pediatrics, The University of North Carolina at Chapel Hill 2331, Chapel Hill, North Carolina, USA; 2 Department of Cell Biology and Physiology, The University of North Carolina at Chapel Hill 2331, Chapel Hill, North Carolina, USA; 3 Department of Pediatrics, The University of Wisconsin-Madison, Madison, Wisconsin, USA; 4 Department of Microbiology and Immunology, The University of North Carolina at Chapel Hill 2331, Chapel Hill, North Carolina, USA; Emory University School of Medicine, Atlanta, Georgia, USA

**Keywords:** Rhinovirus C, CDHR3, airway epithelial cells, pediatric, innate immunity

## Abstract

Rhinovirus C (RV-C) infections during early life are strongly associated with severe wheezing illnesses, lower respiratory tract infections, recurrent hospitalizations, and subsequent asthma development. Cadherin-related family member 3 (CDHR3), the cellular receptor for RV-C, is expressed on the apical surface of ciliated airway epithelial cells, and increased surface expression of CDHR3 has been linked to enhanced viral binding and replication. These observations suggest that age-dependent differences in CDHR3 expression may contribute to the heightened clinical severity observed in young children. In this study, we investigated whether primary pediatric human airway epithelial (HAE) cultures exhibit increased CDHR3 surface expression, enhanced RV-C replication, and/or altered innate immune responses compared to adult HAE. Our findings demonstrate that pediatric and adult HAE have similar amounts of total cellular or surface CDHR3. Consistent with this observation, RV-C replication kinetics and virus-induced innate immune responses were comparable between pediatric and adult HAE. Together, these data indicate that intrinsic epithelial differences in CDHR3 abundance or antiviral signaling are unlikely to fully account for the age-dependent severity of RV-C-associated disease. These results refine our understanding of RV-C pathogenesis and suggest that additional factors, such as undeveloped humoral immunity to RV-C, immune cell interactions, airway microenvironment, or host developmental differences, may play a more prominent role in driving severe disease outcomes in early life.

**IMPORTANCE:**

Rhinovirus C (RV-C) is a clinically significant pediatric respiratory pathogen associated with severe lower respiratory tract disease and an increased risk of asthma development later in childhood. Despite clear age-dependent differences in clinical severity, the underlying biological mechanisms remain poorly defined. Because cadherin-related family member 3 (CDHR3) serves as the cellular receptor for RV-C and is expressed on ciliated airway epithelial cells, variation in epithelial receptor abundance or antiviral responses has been proposed as a potential driver of increased viral burden in young children. To address this, we compared well-differentiated primary airway epithelial cultures derived from pediatric and adult donors. Our findings demonstrate that pediatric epithelial cells exhibit comparable CDHR3 cell surface abundance, RV-C replication dynamics, and virus-induced immune signaling relative to adult cells. These results suggest that factors beyond intrinsic epithelial susceptibility likely contribute to the heightened severity of RV-C disease in early life.

## OBSERVATION

Rhinovirus C (RV-C) has been associated with severe respiratory disease in humans, particularly in children under the age of 3 (1–3). Compared to RV-A or RV-B species, RV-C infections are linked to more serious lower respiratory tract complications, including bronchiolitis, asthma exacerbations, and severe wheezing (4). Notably, RV-C-induced wheezing has been linked to chronic wheezing and subsequent development of asthma (5).

RV-C uses cadherin-related family member 3 (CDHR3) to enter cells (6). Unlike classical cadherin proteins, CDHR3 is not localized to epithelial junctions but is instead expressed on the apical surface of ciliated airway epithelial cells and within the intracellular compartment (7). In airway epithelia, CDHR3 expression is limited to ciliated cells, promoting narrower tropism of RV-C compared to other RV species (6, 8).

Genome-wide association studies of children aged 2–6 years old with recurrent hospitalizations for acute asthma exacerbations identified a single-nucleotide polymorphism (rs6967330) in *CDHR3* that results in a cysteine-to-tyrosine substitution at amino acid residue 529 (C529Y). This variant is associated with a more severe clinical asthma phenotype (earlier age of onset, more hospitalizations per year, and more intensive care unit admissions) in homozygotes and an intermediate phenotype in heterozygous carriers relative to the wild-type (WT) allele, implicating *CDHR3* as an asthma-risk allele (9). Separately, the C529Y mutation has been shown to result in higher levels of CDHR3 on the surface of transformed cell lines and differentiated human airway epithelial (HAE) cells (6–8). Although the mechanism by which increased surface expression of CDHR3 contributes to asthma remains unclear, individuals harboring the risk allele have increased susceptibility to RV-C infection and more severe disease that may lead to repeated airway injury (7, 8).

Young children experience more severe wheezing associated with RV-C infections compared to RV-A or RV-B (3, 10). Further, children 5 years old and younger have more frequent RV-C infections than RV-A infections, with RV-C incidence dropping after this age while RV-A infection incidence remains relatively stable throughout life (11). The biological basis for this age-dependent RV-C susceptibility is not fully understood. One proposed mechanism underlying age-dependent RV-C susceptibility is species-specific differences in the maturation of neutralizing antibody responses, with infection-induced, long-lasting humoral immunity to RV-C developing more rapidly and more frequently than to RV-A during childhood (11). It is also possible that RV-C infections create more durable mucosal memory immune responses. However, given that differential surface expression of CDHR3 is known to mediate increased RV-C susceptibility, we hypothesized that young children may have more surface CDHR3 in their airways that decreases with age. We aimed to compare primary HAE specimens collected from both pediatric and adult donors to test this hypothesis.

**Fig 1:**
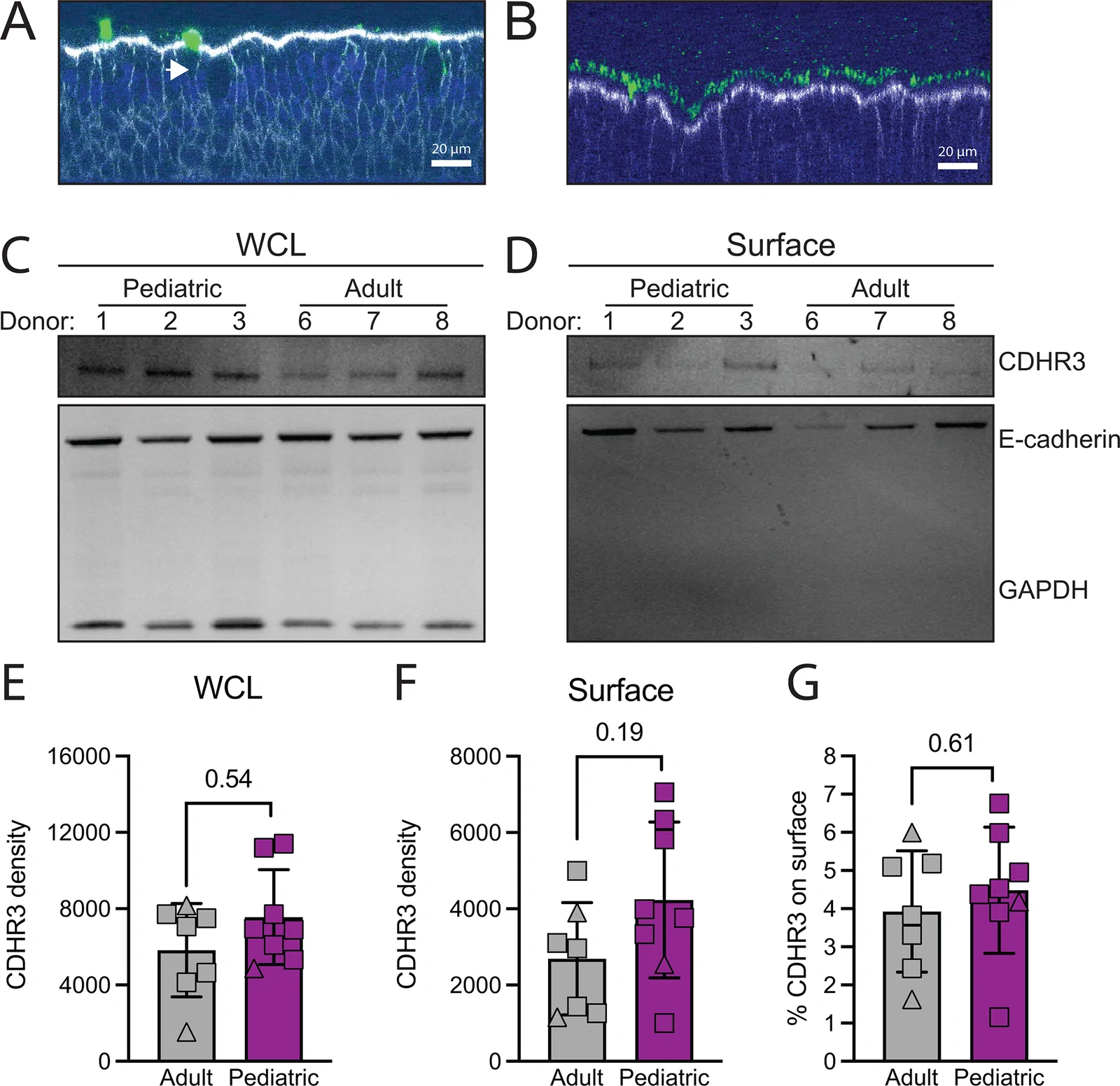
Pediatric HAE exhibit no difference in total or surface abundance of CDHR3 compared to adult HAE. (**A and B**) At 28 days post-differentiation, HAE were fixed and stained for actin (gray), DAPI (blue), and either (**A**) mucin (MUC5AC, in green, arrows pointing to goblet cells) or (**B**) acetylated alpha-tubulin (green, indicating cilia). (**C and D**) Pediatric and adult donor cultures were harvested and immunoblotted for CDHR3, E-cadherin, and GAPDH in the (**C**) WCL or (**D**) surface protein immunoprecipitation fractions. (**E–G**) Densitometry analysis quantifying the abundance of CDHR3 in the (**E**) WCL or (**F**) surface protein immunoprecipitation fractions and (**G**) the percentage of the WCL CDHR3 that was immunoprecipitated in the surface fraction. Squares represent male donors and triangles represent female donors. Each graphed data point represents one culture each from eight pediatric or seven adult donors. Bars represent average values and error bars represent SD. *P* values determined by the Mann-Whitney *U*-test.

To investigate why pediatric populations are at increased risk for severe RV-C infection relative to adult populations, we utilized primary HAE cultures from previously healthy donors that had differentiated for 28 days (Table S1). We selected donors who were younger than 5 years of age at the time of primary airway cell collection for our pediatric cohort, as this is the age below which RV-C infection incidence is higher relative to RV-A (11). All donors were genotyped to determine *CDHR3* alleles at rs6967330. Only donors with homozygous WT alleles were selected, both due to the known effect this would have on CDHR3 surface expression and limited presence of the C529Y allele in our pediatric donors. To validate our HAE culture methods and ensure they generated a differentiated epithelial cell population, we stained for goblet cells (Fig. 1A) and ciliated cells (Fig. 1B). Next, we optimized conditions to biotinylate cell surface proteins so that we could specifically quantify CDHR3, because this protein is also present intracellularly, yet only surface protein would be expected to impact RV-C infection. After confirming the assay worked with fCR3Y HeLa cells that exogenously express CDHR3 (12) (Fig. S1), we biotinylated HAE cell surface proteins, generated whole-cell lysates (WCLs), and then immunoprecipitated the surface proteins with avidin. Surface CDHR3 abundance was then quantified via immunoblot and compared to whole cell protein amounts. E-cadherin but not GAPDH was detected in the cell surface immunoprecipitate, indicating that the biotinylation reagent only contacted cell surface proteins (Fig. 1C and D). We found that pediatric cultures did not have greater levels of total (Fig. 1C and E) or surface (Fig. 1D and F) CDHR3 relative to adult cultures, nor was a higher proportion of the total CDHR3 displayed on the cell surface (Fig. 1G).

**Fig 2:**
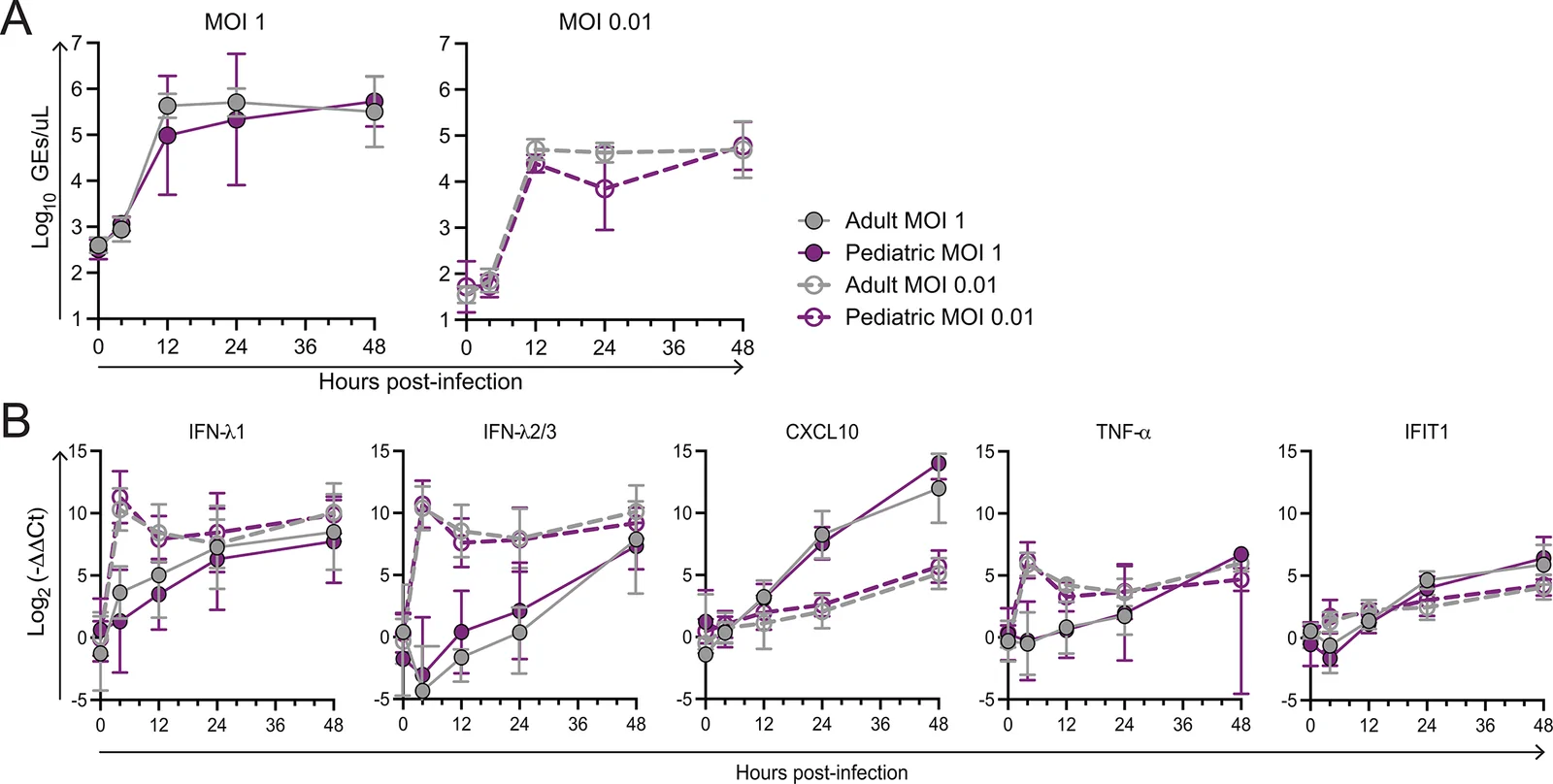
Pediatric HAE demonstrate similar viral growth kinetics and innate immune responses to RV-C challenge compared to adult HAE. Pediatric and adult HAE were infected with RV-C at MOI = 1 or 0.01. RNA was harvested, and (**A**) viral genome equivalents (GEs) or (**B**) expression of IFN-λ1, IFN-λ2/3, CXCL10, TNF-α, and IFIT1 were measured by RT-qPCR at each timepoint. Gene expression data are shown as log_2_ fold change (−ΔΔCt), using β-actin as the housekeeping gene. Each graphed data point represents the geometric mean from eight pediatric or seven adult donors. Error bars represent SD. Statistical comparisons were made between pediatric and adult donors at the same MOI for all time points by unpaired *t*-test with correction for multiple comparisons, and no significant differences were observed.

To determine whether differences in pediatric cultures other than CDHR3 abundance affected RV-C infection dynamics, we performed single-step or multi-step viral growth curves to measure intracellular replication kinetics or cell-to-cell spread, respectively. We noted no difference between the two age cohorts at either multiplicity of infection (MOI) tested (Fig. 2A). For both conditions, RV-C replication spiked from 4 h post-infection (hpi) to 12 hpi, followed by a plateau of detectable virus, consistent with prior observations (13, 14).

Despite similar CDHR3 surface expression and viral replication kinetics, an elevated inflammatory response could also mediate the more severe outcomes of pediatric RV-C infection. We sought to compare the expression of cytokines that have been implicated in host airway epithelia responses to RV-C infection, including IFN-λ, CXCL10, and TNF-a (13, 14). Because RV-C lacks a 5′ cap and therefore does not undergo 2′-O methylation, we also examined the expression of interferon-induced protein with tetratricopeptide repeats-1 (IFIT1), which is generated in response to uncapped RNA (15). We noted no difference between the pediatric and adult cohorts for any cytokine tested at either MOI (Fig. 2B). Both IFN-λ1 and IFN-λ2/3 were robustly induced by 4 hpi at MOI = 0.01, with significantly lower induction observed at MOI = 1. IFN-λ levels plateaued by 12 h post-infection, consistent with the kinetics observed during RV-C infection. CXCL10 levels increased steadily over 48 h, with a markedly higher induction at the higher MOI. While TNF-α had a higher initial response at 4 hpi at lower MOI, similar to IFN-λ1 and IFN-λ2/3, the difference was smaller and quickly diminished by 12 hpi. IFIT1 response to RV-C infection was similar at both MOI tested.

In summary, RV-C infections disproportionately impact young children and are strongly associated with severe lower respiratory tract disease, chronic wheezing, asthma exacerbations, and asthma onset early in life. Despite the larger clinical burden of RV-C in pediatric populations, the mechanisms underlying age-dependent disease disparity remain incompletely understood. Given that RV-C infects ciliated airway epithelial cells by engaging its cellular receptor CDHR3, we aimed to determine whether intrinsic differences in the pediatric airway epithelium contribute to heightened susceptibility to RV-C infection. We hypothesized that increased cell-surface CDHR3 protein expression or altered epithelial antiviral responses in pediatric HAE might drive enhanced viral infection relative to adult HAE. Supporting our hypothesis, a similar study had noted differences in respiratory syncytial virus replication and innate immune response between pediatric and adult HAE (16). However, our data demonstrated that pediatric HAE do not exhibit marked differences in surface expression of CDHR3 protein, susceptibility to RV-C infection, or epithelial-intrinsic immune responses following RV-C infection. Although pediatric HAE displayed a trend towards higher total CDHR3 levels compared to adult HAE, this difference was not statistically significant. Given the limited availability of primary HAE and the resulting constraints on sample size, there may be a true biological difference between pediatric and adult surface CDHR3 expression in the general population that this study is not sufficiently powered to detect. Even if the difference is real, though, surface CDHR3 abundance may not be a dominant determinant of age-dependent RV-C susceptibility as we saw no difference in RV-C replication between the two age groups.

Although the primary airway cells used for our studies were obtained from donors with disparate ages (range of 21–69 years difference between pediatric and adult samples; Table S1), culture conditions may have obscured or eliminated differences that could still be present *in vivo*. While culture conditions were not identical to ours, independent RNA-seq data comparing adult and pediatric HAE cultures showed some age-related variation in global gene expression, but no difference in *CDHR3* (Fig. S2; Table S2) (17). Further, this complementary data set has a balance of female and male donors, whereas our current study was imbalanced (one of eight pediatric donors was female) due to limited primary pediatric airway cells. Therefore, the impact of sex hormones was not captured in our studies.

We noted no difference in RV-C-induced HAE innate immune gene expression. However, age-dependent differences in development of long-lasting humoral immunity, immune cell recruitment and function, maturation of innate and adaptive immune memory responses, or immune-epithelial crosstalk may drive exaggerated or dysregulated inflammatory responses in young children with RV-C infections. These effects, which were not captured by our HAE model, likely contribute to age-related differences in RV-C illness severity.

Altogether, our findings argue against strong epithelial-intrinsic differences in age-dependent RV-C susceptibility and instead point to broader host-contextual factors, operating during a critical window of lung and immune development, as the primary determinants of the disproportionate RV-C burden in early life.

### Cell lines and viruses

WT HeLa cells (ATCC cat# CRM-CCL-2), fCR3Y HeLa cells (12), and WisL (18) cells were grown in Dulbecco’s modified Eagle Medium (DMEM, Gibco cat# 11965092) supplemented with 5% fetal bovine serum and penicillin-streptomycin (100 U/mL–100 µg/mL) (Gibco cat# 15140122). RV-C was transcribed *in vitro* using the pC15-Rz plasmid as previously described (19). Briefly, pC15-Rz was linearized with BstBI at 65°C for 2 h. Linear RNA encoding the RV-C15 genome was transcribed *in vitro* using the RiboMAX Large Scale RNA Production System, T7 (Promega cat# P1300), followed by DNase treatment to remove DNA template. Newly synthesized viral RNA was transfected into WisL cells with Lipofectamine 2000 (Invitrogen cat# 11668027). Transfected cells were incubated at 33°C until virus harvest. Five days post-transfection, infected WisL cells were subjected to three rounds of freeze-thaw cycles. Clarified supernatants were ultracentrifuged for 4 h through a 30% (wt/vol) sucrose cushion at 100,000 × *g*. Viral pellets were resuspended in NTE buffer (120 mM NaCl, 20 mM Tris, and 1 mM EDTA, pH 8.0) with 0.01% BSA (wt/vol) and titers were quantified by qPCR using standard curve generated from *in vitro* transcribed pC15-Rz RNA (19).

### Primary human airway epithelial cell cultures

Seven pediatric and seven adult cryopreserved human airway epithelial (HAE) cells were obtained through the UNC Marsico Lung Institute Tissue Procurement and Cell Culture, procured there under the auspices of UNC IRB #03-1396. Donor #15, an eighth pediatric donor, was obtained from human lungs rejected for transplantation procured through the University of Wisconsin Organ and Tissue Donation (UW OTD), a federally designated organ procurement organization at UW Health, under a protocol reviewed by the University of Wisconsin Institutional Review Board and determined not to constitute human subjects research. Prior to experimentation, HAE cell CDHR3 genotype was determined using RT-qPCR with the Taqman SNP Genotyping Assay kit (ThermoFisher cat# 4351379). After thawing, donor cells were seeded onto bovine type I/III collagen (Advanced BioMatrix cat# 5005) coated plates in Pneumacult Ex-Plus medium (StemCell Technologies cat# 05040) supplemented with hydrocortisone solution (StemCell Technologies cat# 07925) and grown to confluence at 33°C, 5% CO_2_. Next, the basal cells were seeded onto human placental type IV collagen (Millipore Sigma cat# C-7521) coated transwells (12 mm for biotin-immunoprecipitation assays, Corning cat# 3460 or 6.5 mm for RV-C infections, Corning cat# 3470) at a liquid-liquid interface until basal cells reached confluency. The upper chamber medium was then removed from the transwell, exposing the basal cells to the air-liquid interface, with Pneumacult ALI medium (StemCell Technologies cat# 05001) supplemented with heparin (StemCell Technologies cat# 07980) and hydrocortisone solution added to the basal chamber. All HAE were rinsed apically with PBS and given fresh basal medium every other day. Cells were allowed to differentiate at an air-liquid interface for at least 28 days prior to experimentation.

### Cell surface biotinylation and immunoprecipitation

For all immunoprecipitation assays, surface proteins were labeled using the Pierce Cell Surface Biotinylation and Isolation Kit (ThermoFisher cat# A44390) following the manufacturer’s protocol. For HAE surface protein biotinylation, we first treated with 10 mM dithiothreitol (DTT) for 10 min at 37°C to remove mucus present in culture. Cultures were washed 5× with DPBS (Gibco cat# 14190144) after DTT treatment. HAE were exposed to either DPBS or biotin-solution (thiol-cleavable amine-reactive biotinylation reagent) for 30 min at room temperature, gently rocking. After incubations, cells were extensively washed with DPBS and then subjected to cell lysis. A fraction of this cell lysate was collected for WCL analysis. Protein content in the lysate was quantified via the Pierce Bicinchoninic Acid protein assay (ThermoFisher cat# 23250), then 500 µg of each cell lysate was placed inside a column containing NeutrAvidin-coated agarose beads and allowed to incubate at room temperature with over-under mixing for 1 h. After incubation, the columns were washed 5× with wash buffer, and then disulfide bonds were reduced with DTT to cleave bound protein. Eluates were harvested for immunoblot analysis.

### SDS-PAGE and immunoblot

Biotin-immunoprecipitation eluates and WCLs were treated with 4× Laemmli SDS buffer with 5% β-mercaptoethanol and boiled for 5 min at 95°C. Ten micrograms of total protein for WCL gels was loaded into each lane of a 4–20% SDS-PAGE gel. For biotin-immunoprecipitation eluates, 50 µL of each sample was loaded into each lane. All gels were run at 100 V for at least 1 h. After transfer to PVDF membranes, membranes were blocked, then stained with antibodies reactive to E-cadherin (1:1,000, R&D Systems cat# MAB1838), GAPDH (1:1,000, ThermoFisher cat# PA1-987), and/or CDHR3 (1:1,000, Millipore Sigma cat# HPA011218) in blocking buffer overnight at 4°C, rocking gently. Membranes were then washed 5× with DPBS and probed with goat anti-mouse (1:10,000, ThermoFisher cat# 31 430) or goat anti-rabbit (1:10,000, ThermoFisher cat# 31,460) HRP-conjugated secondary antibodies for 1 h. After washing, membranes were developed using ECL western blotting detection reagent (Cytiva cat# RPN3004). Densitometry was performed using the gel analysis tool in ImageJ (v2.14.0/1.54f) to measure area under the curve above background for each CDHR3 band using identically sized regions of interest. When comparing intensity of the WCL to surface CDHR3 bands, the density of both values were adjusted to be from equivalent amounts of initial total protein.

### RV-C infections of HAE

Twenty-eight days post-differentiation, HAE were treated with 10 mM dithiothreitol (DTT) for 10 min at 37°C to remove mucus, then washed 5× with DPBS (Gibco cat# 14190144). Assuming a cell density of 350,000 cells per well, HAE were apically inoculated at an MOI of 0.01 or 1 of RV-C or mock-infected in PneumaCult ALI medium and incubated at 33°C for 4 h. Cells were then washed 3× with DPBS and incubated at an air-liquid interface at 33°C for the remainder of the experiment.

### RNA extraction and quantitative PCR

At the indicated times post-infection, cells from an entire transwell insert were collected in TRIzol (Invitrogen cat# 15596026). Total RNA was extracted (Direct-zol RNA miniprep, Zymo Research cat# R2051) and eluted in water. RNA concentrations were measured, and 500 µg of RNA was used as template to generate cDNA (iScript cDNA synthesis kit, BioRad cat# 1708890). qPCR reactions were performed in duplicate using the primers listed in Table S3. All qPCR reactions were done using *Power*Sybr Green PCR Master Mix (Applied Biosystems cat# 4367659). A standard curve was generated using pC15-Rz plasmid and Ct values converted to genome equivalents for measure of viral RNA quantity (19). For gene expression analysis, −ΔΔCt was calculated using mock-infected controls inoculated at the time of infection. β-Actin was used as a housekeeping gene for all qPCR analyses.

### Immunofluorescence

HAE were fixed with 4% paraformaldehyde 28 days post-differentiation for 30 min at room temperature. Fixed cells were washed with DPBS 3× for 5 min, followed by the addition of permeabilization buffer (0.1% Triton X-100). Cells were washed and then blocked (2.5% goat serum, Vector Laboratories cat# S-1012-50) for 1 h at room temperature. Cells were then incubated with mouse anti-MUC5AC (1:100, ThermoFisher cat# MA1-21907), mouse anti-acetylated alpha-tubulin (1:100, Thermofisher cat# 32-2700), or mouse anti-E-cadherin (1:100, R&D Systems cat# MAB1838). After washing, cultures were incubated with goat anti-mouse IgG Cross-Absorbed Alexa Fluor 488 secondary antibody (1:10,000, Thermofisher cat# A-11001) for 1 h. Cultures were washed and then counterstained with DAPI (Vector Laboratories cat# H-1200-10) and phalloidin 647 (1:10,000, ThermoFisher cat# A22287).

### RNA-seq analysis

RNA-seq data used for this study are available from the Gene Expression Omnibus (GEO) repository at https://www.ncbi.nlm.nih.gov/geo/query/acc.cgi?acc=GSE299837. Normalized gene count matrix files were downloaded and used for the analysis. Donors used for the analysis are listed in Table S2. For each gene, transcript counts (TPM) at days 1, 3, 7, and 14 mock-infected samples were averaged and used as the expression value for each gene. Figures were plotted with R 4.4 and ggplot2 package.

### Statistics

All statistical analyses were performed with GraphPad Prism (v10.6.1). RV-C infection qPCR data represent the geometric mean with seven adult donors and eight pediatric donors. As indicated in the figure legends, statistical differences were calculated using Mann-Whitney *U*-tests, unpaired *t*-tests with Welch’s correction and the False Discovery Rate approach to correct for multiple comparisons, or two-tailed *t*-tests.

## SUPPLEMENTAL MATERIAL

Supplemental tables and figures spectrum.01611-26-s0001.pdf Tables S1 to S3, and Fig. S1 and S2.

ASM does not own the copyrights to Supplemental Material that may be linked to, or accessed through, an article. The authors have granted ASM a non-exclusive, world-wide license to publish the Supplemental Material files. Please contact the corresponding author directly for reuse.
