# Supplementary material for: Pediatric and adult primary human airway epithelia exhibit similar rhinovirus C replication, innate responses, and expression of protein receptor
Source: Microbiol Spectr. 2026 Aug 31;14(10):e01611-26. doi: 10.1128/spectrum.01611-26 (PMC13688799; doi:10.1128/spectrum.01611-26)
Supplement: Supplementary file 1 [file spectrum.01611-26-s0001.pdf]

SUPPLEMENTAL MATERIALS

Title: Pediatric and adult primary human airway epithelia exhibit similar rhinovirus C replication, innate responses, and expression of protein receptor

Authors: Lorellin A. Durnell-Bettis, Ian R. Taylor, You Li, Scott H. Randell, Yury A. Bochkov, James E. Gern, Matthew R. Vogt

Supplemental Table 1: Primary cell donor information.

Donor	CDHR3 529 aa[1]	Age	Designation	Sex	Race/Ethnicity
**1**	C	8 mo	Pediatric	F	Hispanic
**2**	C	7 mo	Pediatric	M	African American
**3**	C	2 yr	Pediatric	M	Caucasian
**4**	C	10 mo	Pediatric	M	African American
**5**	C	1.5 yr	Pediatric	M	Caucasian
**6**	C	21 yr	Adult	M	African American
**7**	C	26 yr	Adult	M	Caucasian
**8**	C	27 yr	Adult	M	Other
**9**	C	23 yr	Adult	F	Caucasian
**10**	C	26 yr	Adult	F	Caucasian
**11**	C	4 mo	Pediatric	M	Caucasian
**12**	C	1 yr	Pediatric	M	Other
**13**	C	37 yr	Adult	M	Caucasian
**14**	C	29 yr	Adult	M	Caucasian
**15**	C	4 yr	Pediatric	M	Caucasian

[1]The WT *CDHR3* allele has cysteine (C) at amino acid (aa) 529. All donors were homozygous for the WT allele.

Supplemental Table 2: Primary cell donor information for RNA-seq analysis.

Donor	CDHR3 529 aa[1]	Age	Designation	Sex	Race/Ethnicity
**1**	C	8 mo	Pediatric	F	Hispanic
**3**	C	2 yr	Pediatric	M	Caucasian
**6**	C	21 yr	Adult	M	African American
**7**	C	26 yr	Adult	M	Caucasian
**8**	C	27 yr	Adult	M	Other
**9**	C	23 yr	Adult	F	Caucasian
**10**	C	26 yr	Adult	F	Caucasian
**11**	C	4 mo	Pediatric	M	Caucasian
**14**	C	29 yr	Adult	M	Caucasian
**083K**	Y	10 mo	Pediatric	M	African American
**048G**	C	5 yr	Pediatric	F	Caucasian
**018I**	Het	2 mo	Pediatric	F	Caucasian
**046M**	Het	4 mo	Pediatric	F	Caucasian
**051K**	C	21 yr	Adult	F	Hispanic
**035P**	C	21 yr	Adult	F	Caucasian

[1]Donors 1, 3, 6, 7, 8, 9, 10, 11, and 14 are the same donors used in our experiments and have the same numbering convention.

[2]The WT *CDHR3* allele has cysteine (C) at amino acid (aa) 529 and the asthma risk allele has a tyrosine (Y). Donors were homozygous for the indicated allele unless designated as a heterozygote (Het).

Supplemental Table 3: Primer sequences used for qPCR assays.

Transcript targeted	Direction	Sequence
**5' UTR RVC**	Fwd	CCTCCGGCCCCTGAAT
	Rev	AAACACGGACACCCAAAGTAGT
**β-actin**	Fwd	CACCATTGGCAATGAGCGGTTC
	Rev	AGGTCTTTGCGGATGTCCACGT
**IFN-λ1**	Fwd	AACTGGGAAGGGCTGCCACATT
	Rev	GGAAGACAGGAGAGCTGCAACT
**IFN-λ2/3**	Fwd	TCGCTTCTGCTGAAGAACTGCA
	Rev	CCTCCAGAACCTTCAGCGTCAG
**CXCL10**	Fwd	GGCATTCAAGGAGTACCTCTCTC
	Rev	TGGACAAAATTGGCTTGCAGGA
**TNF-α**	Fwd	GACAAGCCTGTAGCCCATGT
	Rev	GGAGGTTGACCTTGGTCTGG
**IFIT1**	Fwd	GAAGCAGGCAATCACAGAAA
	Rev	TGAAACCGACCATAGTGGAA

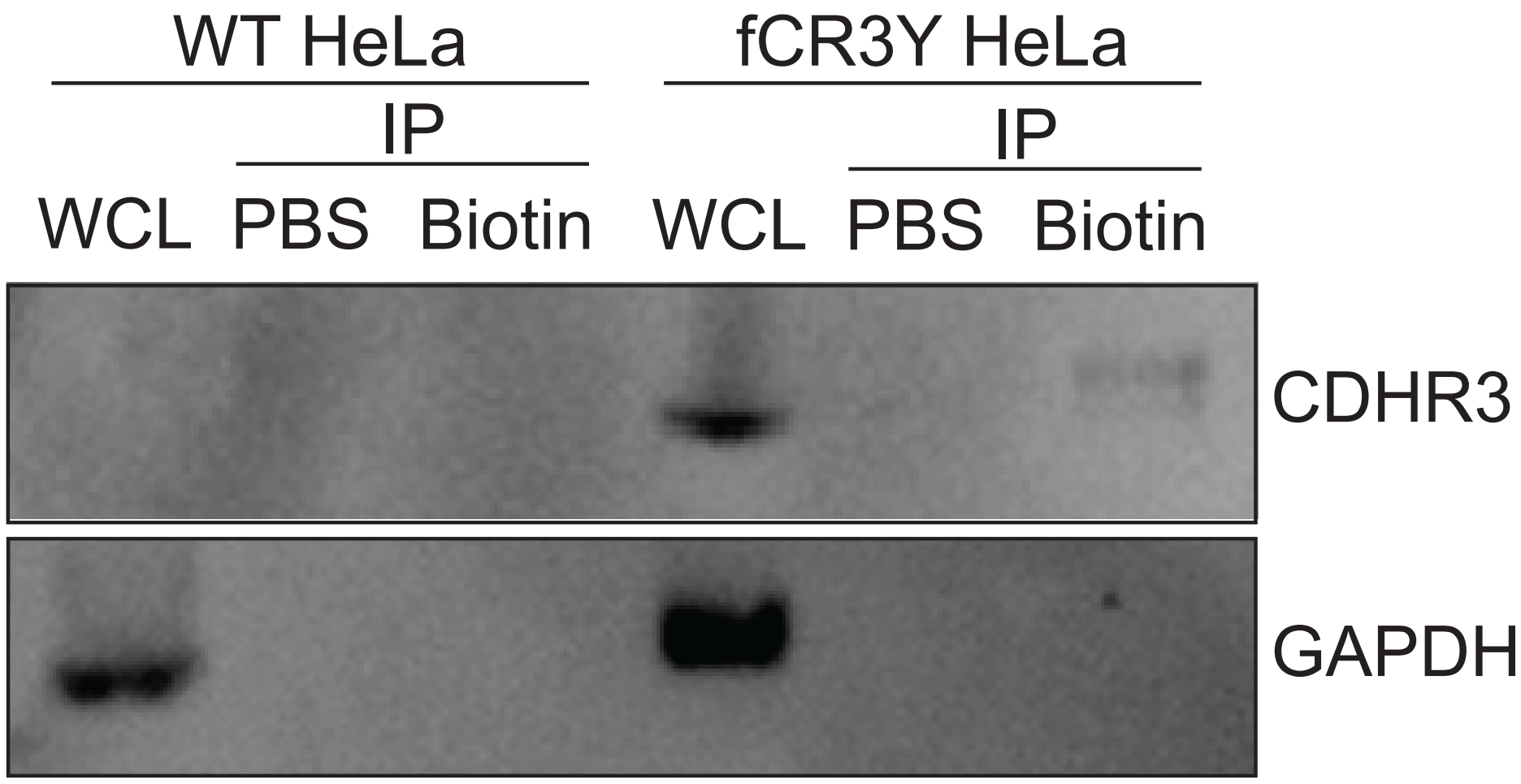

Supplemental Figure 1: Surface protein biotinylation and immunoprecipitation validation in fCR3Y HeLa cells. WT or exogenously CDHR3 expressing fCR3Y HeLa cells were treated with either PBS or biotinylation reagent for 15 minutes. After extensive washing, WCLs were collected and immunoprecipitated with avidin, then CDHR3 or GAPDH were measured by immunoblot.

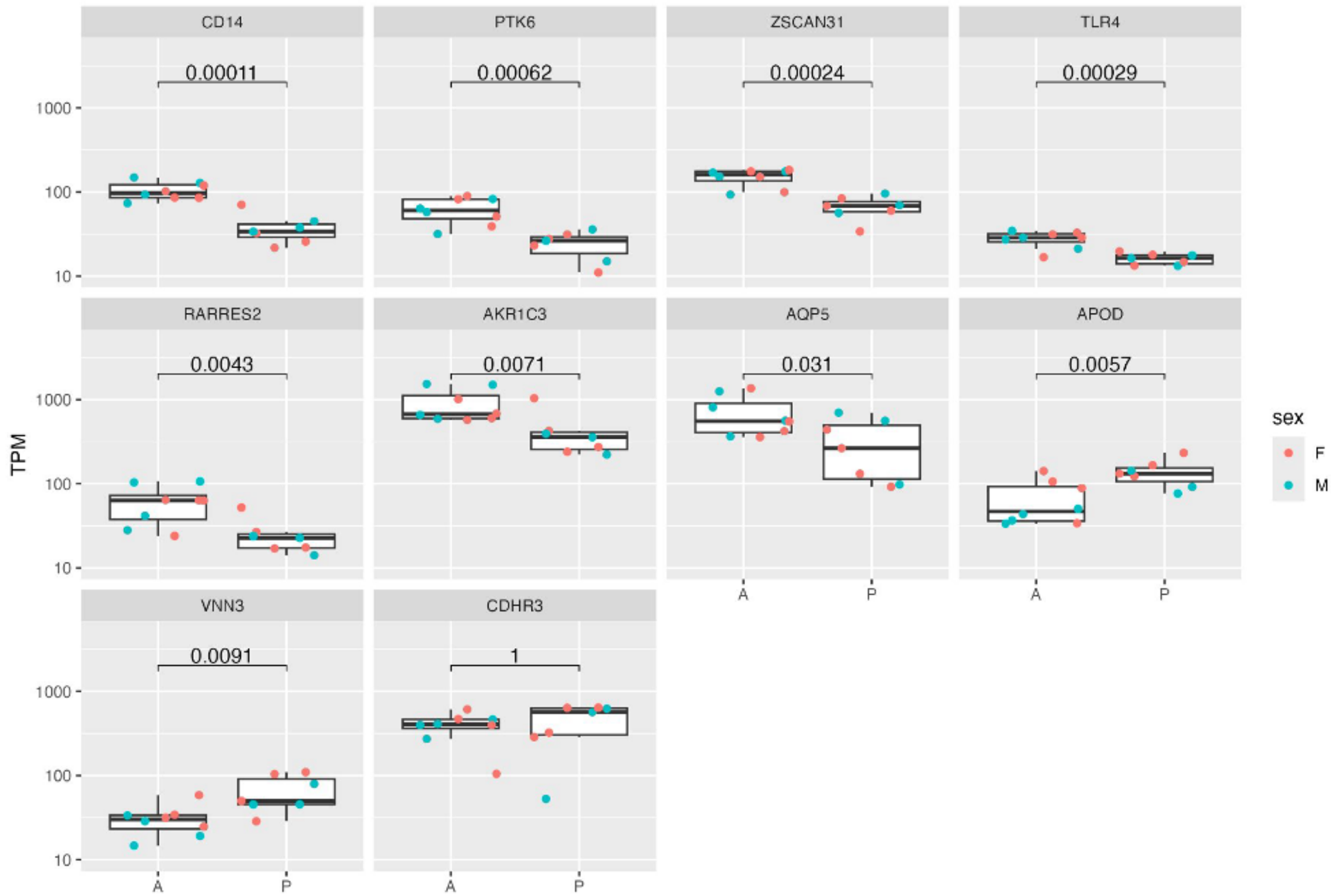

Supplemental Figure 2: Pediatric HAE demonstrate similar global gene expression at baseline compared to adult HAE. Boxplot showing the expression level of indicated genes determined by RNA-seq in HAE cultures from adult (A, n=8, age range 21 years-29 years) and pediatric (P, n=7, age range 2 months-8 years) donors without infection. Genes shown other than *CDHR3* were selected as the top genes that showed any difference between adult and pediatric samples. Data acquired from Dang et al. 2025[1], in which HAE were cultured using different medium. TPM: transcripts per million reads. Colors denote sex. P values calculated by two-tailed t test.
